# Lingual Leiomyomatous Hamartoma in an Adult Male

**DOI:** 10.1155/2018/4162436

**Published:** 2018-08-29

**Authors:** Amanda Phoon Nguyen, Norman Firth, Sophie Mougos, Omar Kujan

**Affiliations:** ^1^UWA Dental School, University of Western Australia, Nedlands, WA 6009, Australia; ^2^Private Practice, OMFSurgery, Cambridge Street, Wembley, WA, Australia

## Abstract

An otherwise healthy 20-year-old male presented with an exophytic, polypoid, yellowish lesion involving the dorsal surface of his tongue, which he reported being present since birth and unchanged. This was removed by surgical excision and diagnosed as a leiomyomatous hamartoma. Histological examination revealed a combination of fibrovascular connective tissue, conspicuous smooth-muscle bundles, adipose tissue, minor salivary gland tissue, blood vessels, lymphoid tissue, peripheral nerves, and normal skeletal muscle. This case is exceptional due to the patient's age, as until now, lingual leiomyomatous hamartomas have been reported almost exclusively in a paediatric population. To our knowledge, this is the eldest age at which a LLH has been reported in the literature. This underscores the need for clinicians to consider this rarely reported entity when considering the radiographic and clinical differential diagnoses for these lesions, both in the paediatric and adult populations. We also present a review of the literature regarding lingual leiomyomatous hamartomas.

## 1. Introduction

The term *hamartoma* is used to describe a nonneoplastic, abnormal, and haphazard overgrowth of conglomerates of mature cells and tissues indigenous to the anatomic site from which it occurs, often with one predominating element [1, 2]. This tumor-like malformation is benign, and most hamartomas are located in the liver, spleen, pancreas, and kidneys [3]. Oral hamartomas are rare [3]. An oral leiomyomatous hamartoma, as the name suggests, is therefore a lesion composed mostly of smooth-muscle tissue. They typically present as smooth, soft, nodular lesions that are present at birth and further develop in the first decade of life [3]. They are usually painless masses without obvious symptoms [1]. In most of the published reports, manifestations have commonly been on the gingiva, tongue, and hard palate, specifically the dorsum of the tongue and the anterior maxillary gingiva or alveolar ridge in the incisive papilla region [1, 3]. Due to its low clinical morbidity and nonspecific symptoms, diagnosis and treatment remains a challenge [4].

Here, we report an unusual case of a lingual leiomyomatous hamartoma (LLH) occurring in a healthy 20-year-old male, treated successfully by surgical excision. We also present the histological and immunohistochemical features as well as a review of the literature. This case is exceptional due to the patient's age, as until now, LLHs have been reported almost exclusively in a paediatric population. To our knowledge, this is the eldest age at which a LLH has been reported in the literature.

## 2. Case Report

**Figure 1:**
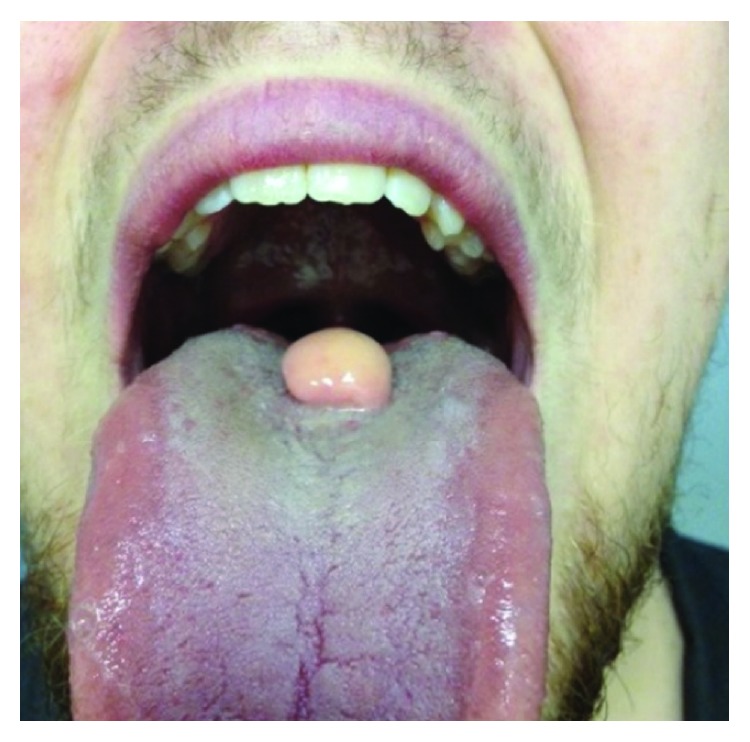
A midline lesion involving the dorsal surface of the patient's tongue.

**Figure 2:**
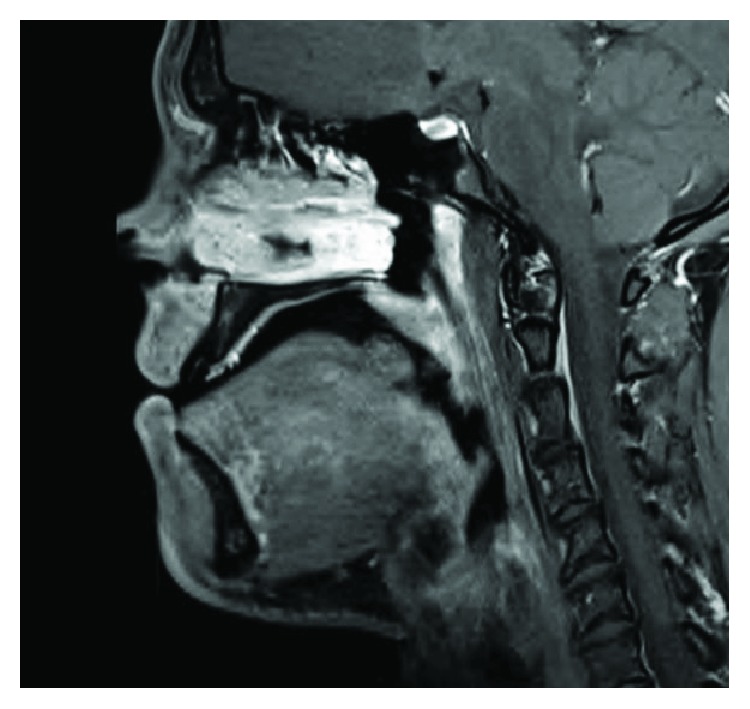
Lingual leiomyomatous hamartoma as seen on magnetic resonance imaging.

A 20-year-old medically fit and healthy male presented for an assessment in preparation for orthognathic surgery. On examination, a 1.5 cm diameter exophytic midline tongue lesion (Figure 1) was noted. This lesion was smooth, regular, and soft to palpation. He reported that this had been present since birth with no change since childhood. A magnetic resonance image (MRI) of his tongue was obtained (Figure 2).

The MRI report described a 1.5 cm protuberant mass arising from the dorsal aspect of the tongue in the midline at the approximate junction of the oral component and base. The imaging suggested that this was in part fatty, probably arising from the submucosa, and is also seen to demonstrate very mild contrast enhancement. There appeared to be intact overlying mucosa and no apparent involvement of the intrinsic tongue muscles. The sublingual space and salivary glands appeared normal.

Thereafter, the mass was surgically excised and submitted for histological examination including haematoxylin and eosin staining and immunohistochemistry.

**Figure 3:**
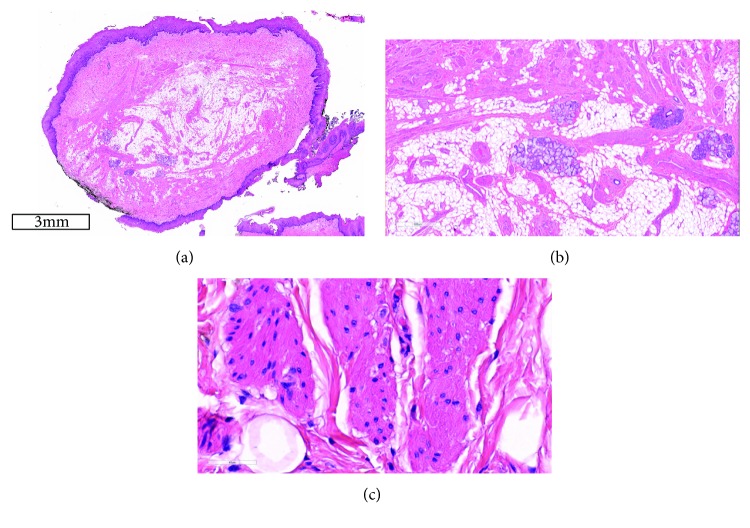
(a) Section of the hamartoma showing fascicles of smooth muscles, fat, and salivary glands, with normal covering epithelium (haematoxylin and eosin, original magnification ×10). (b) Section of the hamartoma showing blood vessels, fascicles of smooth muscles, fat, and salivary glands (haematoxylin and eosin, original magnification ×200). (c) High-power section of the hamartoma showing fascicles of smooth muscles and fat. (haematoxylin and eosin, original magnification ×400).

**Figure 4:**
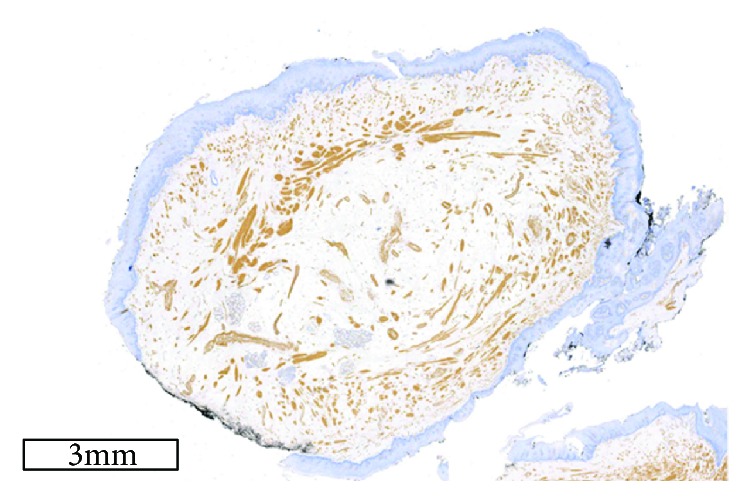
Immunohistochemical findings of a section of the hamartoma showing fascicles of smooth muscles with smooth-muscle actin immunoreactivity (original magnification ×100).

**Figure 5:**
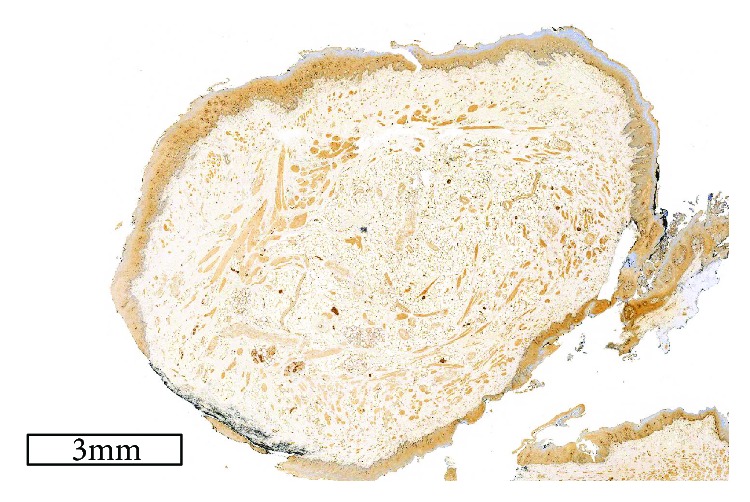
Immunohistochemical findings of a section of the hamartoma showing low S100 immunoreactivity (original magnification ×100).

Histologically, a hamartoma is characterized by a combination of fibrovascular connective tissue, smooth-muscle bundles, skeletal muscle fibers, adipose tissue, salivary tissue, blood vessels, lymphoid tissue, peripheral nerves, and ganglion cells. One type of tissue is determinant in each lesion. In this specimen, microscopy revealed circumscribed nodules covered by stratified squamous epithelium, and interlacing cords of eosinophilic spindle-shaped cells consistent with the smooth muscle within the lamina propria (Figures 3(a)–3(c)). These mature spindle cells with the profile of smooth-muscle cells were determinant of a leiomyomatous hamartoma. Immunostaining for *α*-smooth-muscle actin demonstrated large concentrations of smooth-muscle bundles; however, S-100 was found only in peripheral nerve bundles intermingled with smooth-muscle fibers (Figures 4 and 5). No nuclear atypia, cellular pleomorphism, mitosis, or necrosis was noticed, consistent with the benign and developmental nature of these lesions. Based on these features, a histological diagnosis of a leiomyomatous hamartoma was made.

## 3. Discussion

Hamartomas are pathologically subclassified, depending on the relative abundance of a particular endogenous tissue, and the variants described include vascular, muscle-predominant, adipose tissue-predominant, and intramuscular capillary variants where numerous thin-walled mature capillaries are interspersed between and around muscle bundles [5]. Within the oral cavity, local tissues that might result in hamartomatous growths include odontogenic and nonodontogenic epithelial derivatives, smooth and skeletal muscle, bone, vasculature, nerve, and fat [2]. The borders with the surrounding tissues are typically ill-defined, merging with surrounding tissues [6]. Most hamartomas have been described as pink in colour, ranging in size from 0.1 cm to 2 cm, with a pedunculated, nodular, or polyploidy appearance [7]. These lesions are usually exophytic, although other unusual manifestations such as flat pigmented lesions have been described [6].

LLH most frequently occurs on the anterior palate in the region of the incisive papilla or gingiva and on the midline dorsal tongue, and this predilection may be due to the propensity to dysgenic events in the midline embryonic fusion regions [1]. The most important features of a LLH are its limited growth potential after adolescence and microscopic appearance of unencapsulated admixture of mature cells native to the anatomic location [2]. They are usually painless and relatively small lesions, and most of the published cases involve children (Table 1). They are known to occur most frequently in the first decade of life. In terms of gender, LLH has been reported to occur more frequently in females [3, 18], though the literature regarding this is controversial. In a study by Wushou et al. [4] on 194 cases of head and neck hamartomas, these were more common in males.

Microscopically, LLH should be differentiated from a solid oral leiomyoma and an angiomyoma (angioleiomyoma; vascular leiomyoma); the solid leiomyoma is a true neoplasm of smooth-muscle origin, which is rare in the oral cavity [1].

Clinically, an intraoral solid mass in or near the midline of the maxilla and the tongue, especially in children, should involve LLH as a differential diagnosis. Other lesions that have a similar clinical presentation such as a lipoma, granular cell tumor, choristoma, leiomyoma, fibrous epithelial polyp, and benign mesenchymoma should likewise be considered. As the appearance of LLH can be nonspecific, other lesions such as vascular or lymphatic lesions, mucus extravasation phenomena, reactive or traumatic lesions, benign or malignant neoplasm, and cystic lesions should not be neglected. Additionally, in a paediatric population, hamartomas should be differentiated from lingual thyroid thorough clinical examination, imaging, and histopathological examination.

Given the nonneoplastic nature of hamartomas, complete removal of the tumor mass by wide surgical excision is the accepted treatment of choice. Recurrences have been reported where surgical excision is incomplete. Wushou et al. [4] studied 194 cases of head and neck hamartoma and found that after following the patients for a median of 36 months, 6 patients developed a recurrence. They reported that lesions of less than 6 cm in diameter were well controlled; in contrast, all the recurrences were reported from the larger lesions, of more than 6 cm diameter, which could not be resected completely, especially multiple lesions involving bony tissue. Postoperative radiotherapy was used successfully in one of their recurrent cases [4].

This case is exceptional due to the patient's age, as until now, LLHs have been reported almost exclusively in a paediatric population. This underscores the need for clinicians to gain familiarity and consider this entity when considering the radiographic and clinical differential diagnoses for these lesions, both in the paediatric and adult populations.

## Figures and Tables

**Table 1:** Clinical features of reported cases of lingual leiomyomatous hamartoma.

*N*	First authors	Location on tongue	Age	Sex	Hamartoma type	Congenital?	Associated syndromes
18 cases out of 135	Kreiger et al. [7]	16/18 were dorsal	8 d to 16 y	*M* = 6 *F* = 12	2/8 were neurovascular. 5/18 were SM dominant. 1/18 was fat dominant. 10/18 contained SM and fat.	8	4
						One patient was both.	
1	Fadzilah [8]	Midline posterior mass originating from the tongue base	2 months	M	SM predominant	Yes	No
1	Hanna et al. [9]	Left lateral tongue	3 months	F	SM dominant	Yes	Yes; ectrodactyly-ectodermal dysplasia-clefting syndrome
1	Stamm and Tauber [10]	Base of tongue	Newborn	F	SM dominant	Yes	No
1	Becker et al. [11]	Base of tongue	Newborn	M	SM dominant	Yes	No
1	Takimoto [12]	Base of tongue	6 years	F	SM dominant	Unsure	No
1	Ishii et al. [13]	Base and anterior tongue (4 masses)	4 months	F	SM dominant	Yes	No
1	Goold et al. [14]	Midline dorsum posterior	5 months	M	SM dominant	Yes	No
1	Goldsmith et al. [15]	Posterior tongue	16 months	M	SM dominant	Yes	No
1	Kobayshi et al. [16]	Antero dorsal tongue	3 month	M	SM dominant	Yes	No
1	De la Rosa GarcÍa and Mosqueda-Taylor [17]	Anterior tongue	6 years	M	SM dominant	Yes	No
